# Role of the *DHH1* Gene in the Regulation of Monocarboxylic Acids Transporters Expression in *Saccharomyces cerevisiae*

**DOI:** 10.1371/journal.pone.0111589

**Published:** 2014-11-03

**Authors:** Sandra Mota, Neide Vieira, Sónia Barbosa, Thierry Delaveau, Claire Torchet, Agnès Le Saux, Mathilde Garcia, Ana Pereira, Sophie Lemoine, Fanny Coulpier, Xavier Darzacq, Lionel Benard, Margarida Casal, Frédéric Devaux, Sandra Paiva

**Affiliations:** 1 Centre of Molecular and Environmental Biology (CBMA), Department of Biology, University of Minho, Campus de Gualtar, Braga, Portugal; 2 Centre of Health and Environmental Research (CISA), School of Allied Health Sciences, Polytechnic Institute of Porto, Vila Nova de Gaia, Portugal; 3 Sorbonne Universités, Université Pierre et Marie Curie, UMR7238, Laboratoire de Biologie computationnelle et quantitative, Paris, France; 4 CNRS, UMR7238, Laboratoire de Biologie computationnelle et quantitative, Paris, France; 5 CNRS, UMR8226, Laboratoire de Biologie Moléculaire et Cellulaire des Eucaryotes, Institut de Biologie Physico-Chimique, Paris, France; 6 Sorbonne Universités, Université Pierre et Marie Curie UPMC, UMR8226, Laboratoire de Biologie Moléculaire et Cellulaire des Eucaryotes, Institut de Biologie Physico-Chimique, Paris, France; 7 CNRS, FRE3630, Laboratoire de l’Expression Génétique Microbienne, Institut de Biologie Physico-Chimique, Paris, France; 8 École normale supérieure, Institut de Biologie de l’ENS, IBENS, Paris, France; 9 Inserm, U1024, Paris, France; 10 CNRS, UMR 8197, Paris, France; University Paris South, France

## Abstract

Previous experiments revealed that *DHH1*, a RNA helicase involved in the regulation of mRNA stability and translation, complemented the phenotype of a *Saccharomyces cerevisiae* mutant affected in the expression of genes coding for monocarboxylic-acids transporters, *JEN1* and *ADY2* (Paiva S, Althoff S, Casal M, Leao C. FEMS Microbiol Lett, 1999, 170∶301–306). In wild type cells, *JEN1* expression had been shown to be undetectable in the presence of glucose or formic acid, and induced in the presence of lactate. In this work, we show that *JEN1* mRNA accumulates in a *dhh1* mutant, when formic acid was used as sole carbon source. Dhh1 interacts with the decapping activator Dcp1 and with the deadenylase complex. This led to the hypothesis that *JEN1* expression is post-transcriptionally regulated by Dhh1 in formic acid. Analyses of *JEN1* mRNAs decay in wild-type and *dhh1* mutant strains confirmed this hypothesis. In these conditions, the stabilized *JEN1* mRNA was associated to polysomes but no Jen1 protein could be detected, either by measurable lactate carrier activity, Jen1-GFP fluorescence detection or western blots. These results revealed the complexity of the expression regulation of *JEN1* in *S. cerevisiae* and evidenced the importance of *DHH1* in this process. Additionally, microarray analyses of *dhh1* mutant indicated that Dhh1 plays a large role in metabolic adaptation, suggesting that carbon source changes triggers a complex interplay between transcriptional and post-transcriptional effects.

## Introduction

The cellular metabolism of most yeasts, including *Saccharomyces cerevisiae*, is set to run essentially on glucose. When this yeast encounters harsh conditions in niches deprived from glucose, the ability to transport and metabolize non-fermentable carbon sources is crucial for its survival. In this manner, the uptake of short-chain carboxylic acids across the plasma membrane plays a defining role in the metabolism of yeast cells and in its pH-stasis [1]. Physiological studies, carried out in this baker’s yeast, identified two distinct monocarboxylate proton symporters, strongly repressed by glucose, with different specificities and regulation. A permease involved in the uptake of lactate-pyruvate-acetate and propionate was identified in lactic or pyruvic acid-*S. cerevisiae* grown cells [2], [3], being encoded by *JEN1* [4], whereas, an acetate-proprionate-formate permease was found in ethanol or acetic acid grown cells, with no obvious gene candidate at that time [5], [6]. Later, *ADY2* was identified as the acetate permease encoding gene in *S. cerevisiae* [7].

In an early attempt to identify the genes involved in acetate-proprionate-formate transport, classical genetic studies were carried out. The strain *S. cerevisiae* W303-1A was subjected to UV mutagenesis, in order to obtain mutants affected in the ability to utilize acetic acid, but unaffected on the capacity to grow in ethanol, as the sole carbon and energy source [8]. According to this strategy, it was hypothesised that mutants specifically affected in monocarboxylate permease(s) activity could be found. A mutant clone, exhibiting growth on ethanol, but with pronounced growth defect in a medium with acetic acid, as the sole carbon and energy source, was isolated (Ace8 strain) [8]. Further genotypic characterization of the Ace8 mutant led to the identification of the *DHH1* gene as a most likely candidate for explaining the Ace8 phenotype. Indeed, the transformation of Ace8 cells with a genomic fragment containing *DHH1* restored their capacity to grow on acetate and the deletion of *DHH1* presented slower growth rates than the isogenic wild-type on acetic acid (Paiva, S. 2002 PhD thesis, Fig. S1 in File S1).

*DHH1* encodes a RNA helicase of the DEAD-box subfamily [9], [10]. Several homologs have been described in a broad range of organisms, namely in: *S. pombe*, *STE13* [11], in *Xenopus laevis*, *XP54* [12], in *Drosophila melanogaster*, *ME31B* [13], in mouse, *DDX6* [14], [15], in humans, *DDX6*/*P54*/*RCK* (encoding for an oncoprotein) [14] and in *Caenorhabditis elegans CGH-1* [16]. Dhh1 has been involved in the formation of specific dynamic cytoplasmic loci, the Processing Bodies (P-bodies). P-bodies have been observed in yeasts, insect cells, nematodes and mammalian cells as cytoplasmic foci accumulating translationally silent mRNAs and containing proteins involved in mRNA decay and translation inhibition, including the deccaping enzymes (Dcp1/Dcp2), as well as general activators of deccaping, like Dhh1, Pat1, Lsm1-7 complex, Edc3, the 5′-3′ exonuclease Xrn1, the Non sense mediated mRNA Decay (NMD) regulator Nam7/Upf1, components of the deadenylation machinery, other translational inhibitors, but also translational elongators and ribosomal subunits [17]–[21] (reviewed in [22]). P-bodies were implicated in mRNA decay, mRNA storage and translation repression [23]–[25], miRNA-mediated repression [26], [27], nonsense-mediated decay [28], [29] and viral packaging [30]. In consequence, P-bodies have been proposed to be important players of the cytoplasmic “mRNA cycle”, where normal or aberrant mRNAs having reduced translational rates and enhanced decapping and deadenylation activities are targeted and where they can either be degraded or stored for further translation [31]. However, it should be noted that their role as loci where cytoplasmic mRNA decay actually occurs is still controversial [31]. Namely, many studies have shown that the formation of microscopically detectable P-bodies does not seem to be required for most mRNA degradation pathways and that the mRNAs stored in P-bodies are more stable than the mRNAs found free in the cytoplasm (reviewed in [32]).

Dhh1 plays a fundamental role in regulating the balance between active translation, accumulation in P-bodies and cytoplasmic 5′-3′ decay of mRNAs [33]. Dhh1 physically interacts with the decapping enzyme activator Dcp1, the deccaping enhancers Pat1, Lsm1, Edc3 and the Ccr4-Pop2-Not deadenylase complex [34]–[39]. Mutants deleted for *DHH1*, showed a deficient mRNA decay, longer half-live times of several mRNAs and accumulated capped deadenylated transcripts, indicating that Dhh1 acts as an activator of decapping [35], [40]. More precisely, Dhh1 has been proposed to act on mRNA translation rates [24], [41], [42], based on the reported competition between mRNA deccaping and translation initiation [23], [36]. Former experiments suggested that Dhh1 uses its ATPase activity to release eIF4F complex from the mRNP, or somehow destabilize this eIF4F-mRNA cap complex, and in this manner repress translation initiation with concomitant deccaping stimulation [36]. However, this model has been recently challenged by data indicating that Dhh1 could promote decapping by slowing translation elongation downstream to the initiation step [43] and that its ATPase activity is required for regulating P-bodies dynamics but not translation inhibition [33]. Hence, Dhh1 plays a role in the regulation of several cellular processes [44] including mating [45], filamentous growth [46] and iron deficiency [47]. Moreover, Dhh1 controls the turn-over of the mRNA encoding the decapping enhancer Edc1 [44] and *DHH1* is epistatic on the *DCS1* gene, encoding a decapping enzyme scavenger [48]. Furthermore, *DHH1* has also been involved in mRNA and tRNA nuclear export [41], [49]–[51]. Dhh1 is required for the efficient retrotransposition of Ty1 elements in yeast [52]. Finally, overexpression of *DHH1* also suppresses defects of the mitochondrial Rnase P subunit Rpm2 [53].

In order to elucidate the involvement of this Dead-box RNA helicase in monocarboxylate transport and in the regulation of non-fermentable carbon sources utilization in *S. cerevisiae*, we studied the role of Dhh1 in the expression of the Jen1 permease. We showed that, in the presence of formic acid as sole carbon source, *JEN1* expression is negatively controlled at the post-transcriptional level by a Dhh1-dependent mechanism. The deletion of *DHH1* led to the accumulation of *JEN1* mRNAs which were associated to polysomes but for which no Jen1 protein could be detected, questioning the fact that they are eventually translated. Furthermore, analyses of the wild-type and *dhh1* mutant cell transcriptomes evidenced the broad involvement of this RNA helicase in the control of various cellular pathways.

## Materials and Methods

### Yeast strains, plasmids and growth conditions

*S. cerevisiae* strains used in this work are listed in table 1 and the plasmids in table 2. The cultures were maintained on plates of yeast extract (1%, w/v), peptone (1%, w/v), glucose (2%, w/v) and agar (2%, w/v). Yeast cells were grown in YNB glucose 2.0% (w/v), supplemented with adequate requirements for prototrophic growth. Carbon sources were glucose (2%, w/v), lactic acid (0.5%, v/v, pH 5.0), acetic acid (0.5%, v/v, pH 6.0), formic acid (0.5%, w/v, pH 5.0) and propionic acid (0.5%, v/v, pH 5.0). Solid media were prepared adding agar (2%, w/v) to the respective liquid media. Growth was carried out at 30°C, both in solid or liquid media. The cells were also directly grown in rich media, YP lactic acid 0.5% pH 5.0, or YP acetic acid 0.5% pH 6.0. YNB glucose-containing media was used for growth of yeast cells under repression conditions. For derepression conditions glucose-grown cells were harvested during the exponential phase of growth, centrifuged, washed twice in ice-cold deionised water and cultivated into fresh YNB medium supplemented with a carbon source of choice.

**Table 1:** *S. cerevisiae* strains used in this work.

Strains	Genotype	Reference
W303-1A	MATa *ade2-1*; *leu2–3*, *112*; *his3–11*, *15*; *trp1*Δ*2*; *ura3-1; can 1–100*	[90]
ACE 147	W303-1A; *dhh1*Δ::*kan*MX4	Paiva S., 2002 PhD thesis
*jen1*	W303-1A; *jen1*Δ*::HIS3*	(Casal et al. 1999)
BLC 491-U2	MATa ura3–52 *JEN1::GFP-kan*MX4	[55]
NV1	ACE 147; *dhh1*Δ::*hph*MX4	This work
NV2	NV1; *JEN1::GFP-kan*MX4	This work
ACE 145	W303-1A: *JEN1::GFP-kan*MX4	Paiva S., 2002 PhD thesis
BY 1 (YJL124c)	BY4742; MAT alpha; *his3*Δ*1*; *leu2*Δ*0*; *lys2*Δ*0*; *ura3*Δ*0*; YJL124c::*kan*MX4	Euroscarf
BY 2 (YCR077c)	BY4741; MAT a; *his3*Δ*1*; *leu2*Δ*0*; *met15*Δ*0*; *ura3*Δ*0*; YCR077c::*kan*MX4	Euroscarf
BY 3 (YMR080c)	BY4741; MAT a; *his3*Δ*1*; *leu2*Δ*0*; *met15*Δ*0*; *ura3*Δ*0*; YMR080c::*kan*MX4	Euroscarf
BY 4 (YOR076c)	BY4741; MAT a; *his3*Δ*1*; *leu2*Δ*0*; *met15*Δ*0*; *ura3*Δ*0*; YOR076c::*kan*MX4	Euroscarf
MAR 5	W303-1A: *JEN1::GFP-hph*MX4	This work
MAR 6	MAR 5; *lsm1*Δ	This work
MAR 7	MAR 5; *pat1*Δ	This work
MAR 8	MAR 5; *nam7*Δ	This work
MAR 9	MAR 5; *ski7*Δ	This work
MAR 14	W303-1A; *lsm1*Δ	This work
MAR 15	W303-1A; *pat1*Δ	This work
MAR 16	W303-1A; *nam7*Δ	This work
MAR 17	W303-1A; *ski7*Δ	This work

**Table 2:** Plasmids used in this study.

Plasmids	Source or references
pT12	[4]
pPDA1	Andrade, R. (This work)
pAG32	[54]

### Strains construction

The yeast strains, the plasmids and the primers used in this work are listed respectively in Tables 1, 2 and 3. The mutant strain, *dhh1*, carrying a *dhh1::kan*MX4 locus, was transformed with the hygromycin resistance gene *hph*MX4 resulting in a marker switch producing the *dhh1::hph*MX4 *locus* [54]. The same procedure was made for the strain W303-1A: *JEN1::GFP-kan*MX4 resulting in the strain W303-1A: *JEN1::GFP-hph*MX4. The *S. cerevisiae* strain BLC 491-U2, was used to amplify the genetic chimaera, *JEN1::GFP-kan*MX4, using the primers W303-1A forward and W303.1A reverse [55]. The *dhh1::hph*MX4 strain was subsequently transformed with the 2.8 Kb *JEN1::GFP-*kanMX4 PCR product resulting in strain NV2. Transformed cells were grown in YPD media, for 4 hours, and spread on YPD plates, containing 200 mg L^−1^ of Geneticin (G418 from Invitrogen) and 300 mg L^−1^ of Hygromycin (Hygromycin B from Invitrogen). The obtained transformants were confirmed by analytical PCR, with primers A1 and GFP rev [56].

**Table 3:** Primers used in this work.

Primers	Sequence
W303-1A forward	GATTTGTCCTCTCCTGTTATGAAG
W303-1A reverse	ATCTTGCTAGTGTTAACGGCTGTTA
A1	GGCCTATCCAAGGATGCTGTC
GFP_rev	AACATCACCATCTAATTCAAC
A LSM1	ACCGTATGGGTCTTTGATACACTTA
D LSM1	GGTCTACTGAGCTTACAATAGCAGC
A PAT1	CATTTTAATGGAGTAATTGTCCTGG
D PAT1	TCAAATAGTCGTTCTCCTCAAGTTC
A NAM7	TTTAGTATCATCAGTTTCCCTTTGC
D NAM7	TGATTAAACGAGCTTTCAATTTTTC
A SKI7	GTGATTTTCTACAATCAAACAACCC
D SKI/	GAAATTCTCAATGGCTACTTTACGA
K2	CGATAGATTGTCGCACCTG
K3	CCATCCTATGGAACTGCCTC

To obtain the *lsm1*, *pat1*, *nam7* and *ski7* mutants in the W303-1A background, strains BY 1 to 4 were used to amplify the corresponding deletion cassettes, using the respective A and D primers. The strains W303-1A and W303-1A: *JEN1::GFP-hph*MX4 were then transformed with this PCR product. The transformed cells were grown in YPD media for 4 hours and spread on YPD plates, containing 200 mg L^−1^ of Geneticin and/or 300 mg L^−1^ of Hygromycin.

Cloning and PCR amplification analyses were performed as previously described [57].

### Transport assays

YNB glucose-containing media was used for growth of yeast cells under repression conditions. For derepression conditions glucose-grown cells were harvested during the exponential phase of growth, centrifuged, washed twice in ice-cold deionised water and cultivated into fresh YNB medium supplemented with a carbon source of choice. Cells were harvested by centrifugation, washed twice and resuspended in ice-cold deionized water to a final concentration of 20–40 mg dry weight/ml. Conical centrifuge tubes containing 30 µl of 0.1 M KH_2_PO_4_ buffer at pH 5.0 and 10 µl of the yeast suspension were incubated for 2 min at 26°C. The reaction was started by the addition of 10 µl of an 2 mM aqueous solution (saturation concentration) of 4000 d.p.m./nmol of radiolabeled [^14^C] lactic or [^14^C] acetic acid (sodium salt; GE Healthcare) at pH 5.0. The reaction was stopped by dilution with 5 ml of ice-cold water. The reaction mixtures were filtered immediately through GF/C membranes (GE Healthcare) and the filters were washed with 10 ml of ice-cold water and transferred to scintillation fluid (Opti-Phase HiSafe II; Pharmacia LKB). Radioactivity was measured in a liquid scintillation spectrophotometer (Tri-Carb 2200 CA; Packard Instrument Co.) equipped with a d.p.m. correction facility. For nonspecific adsorption of [^14^C] lactic acid was added at time zero after the cold water. All experiments were repeated at least three times, and the data reported represents average values. Data obtained is represented as the mean ± SD of triplicate measurements.

### Microscopy

*S. cerevisiae* living cells were examined with a Leica Microsystems DM-5000B epifluorescence microscope with appropriate filter settings. Images were acquired with a Leica DCF350FX digital camera and processed with LAS AF Leica Microsystems software.

### RNA analysis

Total RNA was isolated using the standard hot acidic phenol protocol. In a 1.5% (w/v) agarose/MOPS/formaldehyde gel, samples of 20 µg RNA were electrophorised and blotted onto a Hybond- N+ membrane [58]. An internal fragment of 844 bp, obtained by the digestion of the pT12 plasmid (Table 2) with the restriction enzymes *Nco*I and *Pst*I, was ^32^P-labelled and used as a *JEN1* probe. As internal control RNA of *PDA1* was also used. For mRNA relative half-life times (t ½ mRNA) determination, inhibition of transcription was accomplished by the addition of 1,10-phenantroline (0.1 mg.ml^−1^) for 0, 4, 10 and 20 minutes [59]. Real time quantitative RT PCR experiments were conducted as previously described (Garcia et al., MBC 2007) using primer pairs specific of *JEN1* (sequences), *ACT1* (sequence) or *SCR1* (sequences). Relative half-live times were determined by measuring the ratio between amounts of *JEN1* mRNA and those of SCR1 at each time point and applying, a linear regression equation to the Log (*JEN1/SCR1*) = f(t) function. The calculation of the slope directly gave access to the relative half-life of *JEN1* in the mutant and the wild-type strain. The reported half-live times represent a mean value obtained from three experiments performed on independent biological samples.

### Polysome gradients

*dhh1* mutant cells were grown in YNB glucose media until they reached an optical density of 0.8. Then, they were washed twice in sterile water and transferred into an equivalent volume of either YNB lactic acid or YNB formic acid media. After 5 hours, cycloheximide was added to the cells at a final concentration of 100 µg/ml. After 5 minutes of incubation on ice, the cultures were centrifuged 5 minutes at 3000 g, washed once with sterile water and a second time with lysis buffer (Tris-HCl pH 7.4 20 mM, NaCl 50 mM, MgCl_2_ 5 mM, DTT 1 mM, cycloheximide 100 µg/ml). After the last centrifugation, the cell pellet was resuspended in 800 µl of lysis buffer including 1X protease inhibitor cocktail (Roche). An equivalent volume of glass beads were added and the cells were disrupted by vortexing 6 times 30 seconds, at 4°C). The supernatant was collected and centrifuged 5 minutes at 5000 g (4°C). The supernatant was cleared by two rounds of centrifugation 10 minutes at 12000 g (4°C). The final supernatant was stored at −80°C after adding 10% glycerol. About 600 µl of this solution was loaded on a 10%–50% sucrose gradient, centrifuged at 39000 g for 3 hours and 0.5 ml fractions were collected in 2 ml tubes using a Retriever 500 (ISCO) fraction collector and a Type 11 Optical unit (ISCO) with 254 nm filters. The RNAs contained in the fractions were precipitated by added 50 µl of ammonium acetate 3 M pH 5.3 and 1.2 ml of absolute ethanol. The mixtures were stored overnight at −20°C, and then they were centrifuged for 20 minutes at 15000 g (4°C). The pellets corresponding to the polysomes fractions were resuspended with the same 100 µl of water. The pellets corresponding to the other fractions were pooled in another 100 µl of water. The RNA was then purified and cleaned up using the RNA easy midi kit (Qiagen), following the supplier’s recommendations. The RNA was quantified by spectrometry and 0.5 µg (for lactic acid) or 1.5 µg (for formic acid) were used for reverse transcription and real-time quantitative PCR analyses using the *JEN1* and *ACT1* primer probes as described above.

### Microarray analysis

Detailed protocols are described at http://www.transcriptome.ens.fr/sgdb/protocols/. The *S. cerevisiae* microarrays used are fully described in Array express (www.ebi.ac.uk/microarray-as/aer/entry; accession number A-MEXP-337). The microarray experiments were conducted as previously described [60]. Raw data were normalized using global lowess followed by print-tip median methods, with background removal, as implemented in Goulphar [61]. Experiments were carried out 2 times, with dye swapping. The microarray data are available in Table S2 and fully available at the GEO database (accession number: GSE60983). The statistical significance of the expression variations measured was addressed by using the TMEV version of SAM with a FDR of 5%, a S0 calculated by the Tusher method and using the exact number of permutation [62]–[64]. Only genes that passed the SAM filter and had an average log2 of ratio above 0.9 in glucose or in formic acid were considered as significantly changing their expression in the mutant compared with the wild type. These genes were listed in Table S1. Global functional analyses were performed using FUNSPEC [65].

## Results

### *JEN1* expression profile

**Figure 1:**
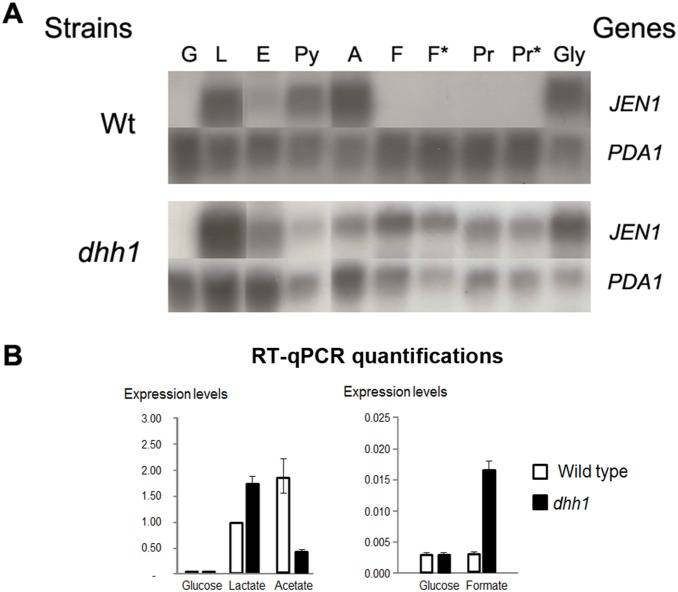
*JEN1* expression profile. A- Transcription analyses of *JEN1* in *S. cerevisiae* W303-1A wild-type and *dhh1* cells. Total RNA was isolated from YNB Glucose 2% (w/v) grown cells, collected at mid exponential phase, and after induction for 4 or 6 hours in different non-fermentable carbon sources: G – glucose; L – lactic acid (4 hours); E – ethanol (4 hours); Py – pyruvic acid (4 hours); A – Acetic acid (4 hours); F – formic acid (4 hours); F* – formic acid (6 hours); Pr – propionic acid (4 hours); Pr* – propionic acid (6 hours); Gly – glycerol (4 hours). An internal *JEN1* fragment was used as probe. *PDA1* was used as a reference, for relatively constant transcription. B- RT-QPCR analyses of *JEN1* mRNA expression levels in wild type and *dhh1* cells grown on glucose, lactic acid, acetic acid or formic acid. The *JEN1* expression levels indicated here are relative to *SCR1*, a RNA pol III transcript which is not supposed to be sensitive to the deletion of *DHH1*. A different control was used for Q-PCR (*SCR1*) and northern blot (*PDA1*) analyses to ensure that the measured effects were not a bias coming from the control. The levels of *JEN1* mRNAs measured in wild-type cells in lactic acid were used as a reference and arbitrarily set up at a value of 1. The experiments were performed three times on biologically independent samples.

W303-1A and *dhh1* cells were grown in different carbon and energy sources, in an effort to clarify the mechanisms involved in the expression regulation of *JEN1* by *DHH1*. The expression pattern of *JEN1* was studied by Northern blot analyses (Fig. 1A). In the wild-type strain, *JEN1* was highly expressed in lactate, acetate and glycerol, poorly expressed in ethanol and almost totally absent in glucose, formic acid and propionic acid. In the *dhh1* strain, the *JEN1* expression profile was similar to the wild-type with the notable exceptions of pyruvic, acetic, formic and propionic acids. In the presence of formate and propionate, the *JEN1* mRNA largely accumulated in the mutant, indicating either a derepression of *JEN1* transcription or a stabilization of *JEN1* mRNA in this strain as compared with the wild-type. In the presence of pyruvate and acetate, an opposite behavior was observed: the *JEN1* mRNA was less abundant in the *dhh1* strain than in the wild-type. *JEN1* expression was also quantified by real-time quantitative PCR in wild-type and *dhh1* cells grown in glucose, lactic acid, acetic acid or formic acid, which confirmed the conclusions taken from the northern blots (Fig. 1B). These results show that *DHH1* regulates the expression of *JEN1* at the mRNA level, pointing to an involvement of this RNA helicase in the regulation of monocarboxylic acids utilization, in *S. cerevisiae*.

### Involvement of other players in the mRNA degradation pathways

**Figure 2:**
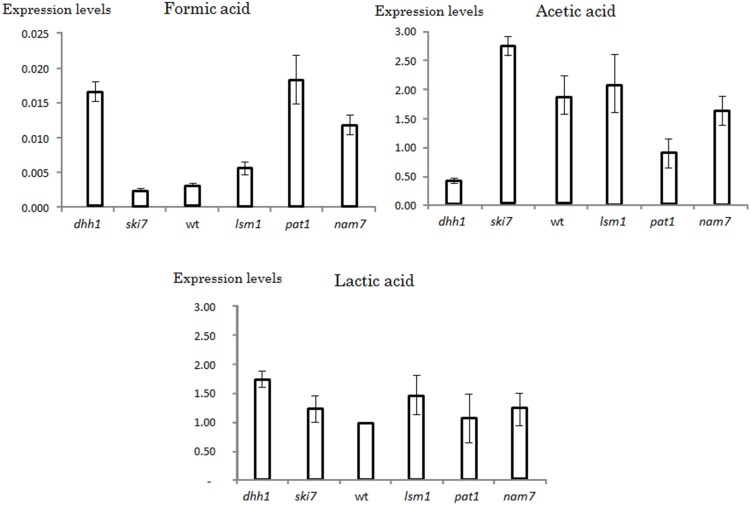
Real time quantitative RT-PCR analyses of *JEN1* mRNA steady states in different carbon sources and genetic contexts. Wild-type, *dhh1*, *pat1*, *lsm1*, *nam7* and *ski7* strains were grown in formic, acetic or lactic acid as sole carbon sources. The levels of *JEN1* mRNA were measured by real time PCR and normalized by the levels of *SCR1* RNA. The levels of *JEN1* mRNAs measured in wild-type cells in lactic acid were used as a reference and arbitrarily set up at a value of 1. The experiments were performed three times on biologically independent samples.

To further investigate the mechanisms by which Dhh1 controls *JEN1* expression, we measured the *JEN1* mRNA levels in cells mutated for genes involved in various aspects of cytoplasmic mRNA degradation: decapping activation (*pat1* and *lsm1*), 3′-5′ exosome-mediated degradation and the non-stop decay (*ski7*) and non sense mediated decay (*nam7*). The inactivation of *PAT1* or *NAM7* led to an accumulation of *JEN1* mRNA in formic acid of 6 and 4 fold, respectively, as compared with the wild-type. This effect was similar to what was observed in the *dhh1* strain (Fig. 2, left panel). The *lsm1* strain exhibited an increase in *JEN1* mRNA, which was significant, but lower for the one observed for *pat1* and *nam7* strains (about 2 fold). In contrast, the deletion of *SKI7* had no effect on *JEN1* expression in these growth conditions. With acetic acid as the sole carbon source, the deletion of *DHH1* or *PAT1* led to a significant decrease of *JEN1* expression of 5 and 2 fold, respectively (Fig. 2, right panel). The other mutants examined showed no significant differences as compared with the wild-type. Finally, in lactic acid, the mutations tested had few effects on *JEN1* mRNA levels (Fig. 2, lower panel). These results suggested contrasted roles of Dhh1, Pat1 and Nam7 on *JEN1* expression, which largely vary depending on the carbon source. We further investigated the regulation of *JEN1* in formic acid.

### Decay of *JEN1* mRNA in the *S. cerevisiae dhh1* and *nam7* strains

**Figure 3:**
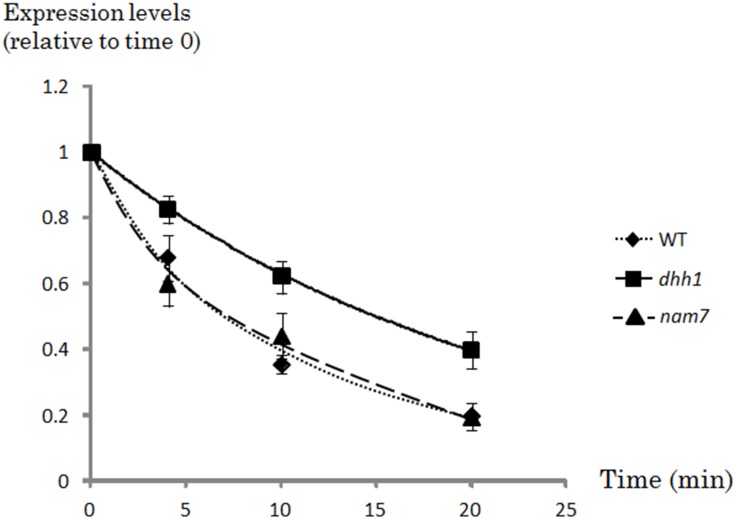
Real time quantitative RT-PCR analyses of *JEN1* mRNA stability in YP lactic acid-grown wild-type, *nam7* and *dhh1* mutant cells. Mutant and wild-type cells were grown in YP lactic acid and collected immediately before (time zero) or 4, 10 or 20 minutes after the addition of 1,10-phenantroline (0.1 mg/ml). The expression levels are expressed relatively to the *SCR1* expression level used as a control. These relative expression levels were set to one at time zero for both strains, in order to normalize differences in mRNA levels between the two strains at the beginning of the experiment. Squares represent the values obtained for the *dhh1* mutant, triangles represent the values obtained for the *nam7* mutant and diamonds represent the values obtained for the wild-type. The experiments were performed three times on biologically independent samples.

The relative stability of *JEN1* mRNA was analysed in the *S. cerevisiae* wild-type strain and in a *dhh1* or *nam7* genetic background. A pulse of 1,10-phenanthroline (0.1 mg/ml) was added to YP lactic acid-grown cells (*JEN1* inducing conditions) to stop transcription. RNA samples were prepared 0, 4, 10 and 20 minutes after the pulse. *JEN1* mRNA levels were measured using real time quantitative PCR. The results were normalized to the signals obtained for the *SCR1* mRNA (a stable RNA pol III transcript) and time zero was used as a reference to normalize for RNA steady state differences between wild-type and *dhh1* strains before the phenanthrolin pulse (Fig. 3). The relative half-live times (t ½ mRNA) of *JEN1* mRNA were calculated in each strain. This relative half-life increased by two fold in the *dhh1* mutant as compared with the wild-type and nam7 mutants (15+/−1.05 minutes in *dhh1* mutant, 8.5+/−0.9 and 8.7+/−1.2 minutes in the wild type and *nam7* mutant, respectively) (Fig. 3). These results suggest that *dhh1* actively participates to the regulation of the stability of *JEN1* mRNA in formic acid. In contrast, the increase of *JEN1* mRNA seen in the *nam7* mutant is not related to a higher stability, suggesting that Dhh1 and Nam7 act at different levels in the regulation of *JEN1*.

### Activity of monocarboxylic acids transporters in *S. cerevisiae dhh1* strain

**Figure 4:**
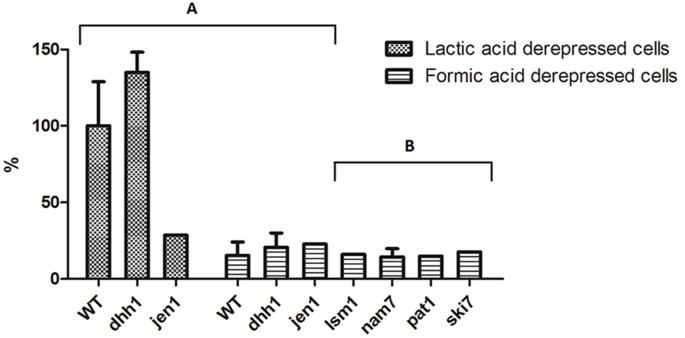
Transport activity of lactic acid in *S. cerevisiae* W303-1A strains: wild-type, *dhh1*, *jen1* (A), *lsm1*, *ski7*, *pat1* and *nam7* (B). The results are percentages of initial activities of 2 mM [^14^C] lactic acid uptake, pH 5.0. Cells were grown in YNB glucose and derepressed in YNB lactic acid or YNB formic acid. Wild-type and *dhh1* YNB lactic acid derepressed cells were used as a control.

In order to determine whether *JEN1* mRNAs detected in *dhh1*, *pat1* and *nam7* mutant strains grown in formic acid was being translated to a functional protein, the uptake of 2 mM of labelled lactic acid pH 5.0, was assessed in wild-type and mutant cells grown on glucose and shifted to YNB formic acid 0.5%, pH 5.0, for 4 hours (Fig. 4B). As a control, wild-type and *dhh1* mutant cells were grown in glucose and shifted to YNB lactic acid 0.5%, for 4 hours and no significant differences were observed in the uptake of labelled lactic acid (Fig. 4A), in contrast to what was found for *jen1* mutant, where no active transport was observed. In formic acid, no lactate transporter activity was observed in the wild-type cells, which is in accordance with the fact that no mRNA expression was found in these conditions. In *dhh1*, *pat1* and *nam7* mutants, although the *JEN1* mRNA was accumulating, no active lactate transport could be detected (Fig. 4B), suggesting that the accumulation of *JEN1* mRNAs in the absence of *DHH1* does not lead to detectable Jen1 activity.

### Jen1 protein is undetectable in the *dhh1*, *pat1* and *nam7* mutant strains grown in formic acid

**Figure 5:**
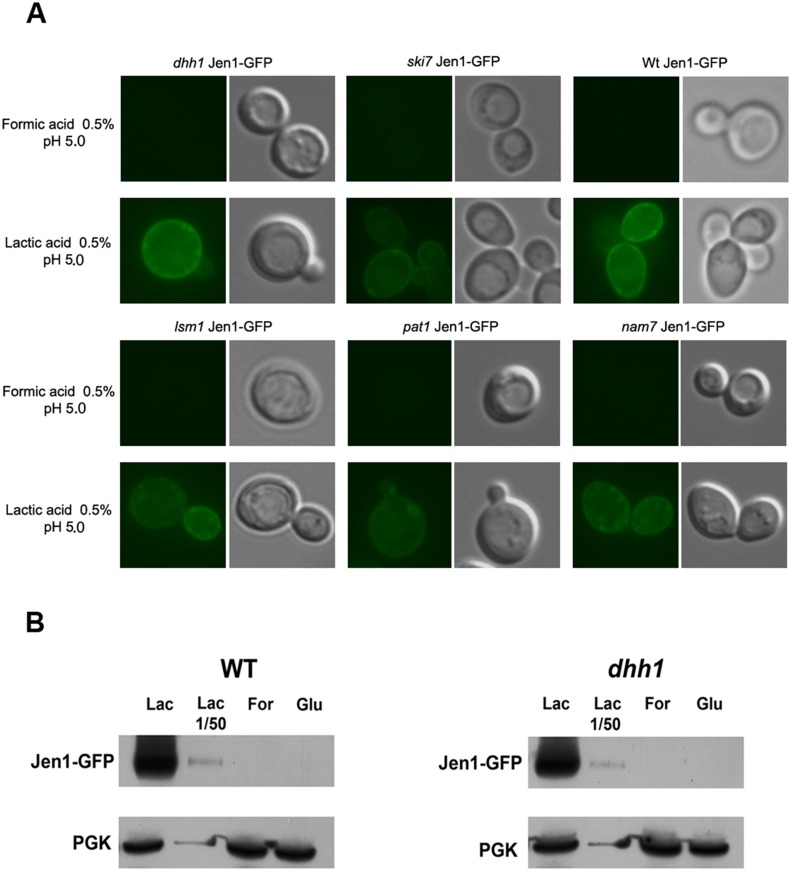
Jen1 protein expression. A - Subcellular localization of *Jen1::GFP* in *S. cerevisiae* living cells. Wild-type, W303-1A, *dhh1*, *lsm1*, *pat1*, *nam7* and *ski7* mutant cells harboring *Jen1*::GFP were used to follow Jen1 expression after growth in YNB glucose, and derepression for 4 hours in YNB lactic 0.5%, pH 5.0, or YNB formic acid 0.5%, pH 5.0. B – Total protein extracts from wild type and *dhh1* mutant cells harboring *Jen1*::GFP were used to follow Jen1 expression after growth in YNB glucose, and derepression for 4 hours in YNB lactic 0.5%, pH 5.0, or YNB formic acid 0.5%, pH 5.0 and immunoblotted with the indicated antibodies. We add a sample of extracts from lactic acid 50 fold diluted.

To try to detect Jen1 protein in formic acid, cells of the wild-type and the *dhh1*, *nam7*, *pat1*, *lsm1* and *ski7* (Table 1) mutant strains, harboring a gene encoding a Jen1::GFP chimera in their genome, were grown in YNB formic acid or in YNB lactic acid (positive control) for 4 hours. Cells were harvested and equal volumes of cell suspension were resuspended in low-melt agarose (1.0%, w/v), and observed by epifluorescence microscopy. In lactic acid, the fluorescence was unambiguously localized to the plasma membrane in all tested cells as previously described for the wild-type strain [55] (Fig. 5A). The same experiments were conducted with cells grown in formic acid as sole carbon and energy source for 4 and 6 hours. In these conditions, there was no fluorescence of Jen1::GFP in any of the strains tested (Fig. 5A). Again, this result was expected for the wild-type, in which almost no *JEN1* mRNA is present, but not for *dhh1*, *pat1* or *nam7* mutants, in which significant amounts of *JEN1* mRNA were detected by Q-PCR and northern blots. However, this may just be a problem of detection sensitivity, because, even in the *dhh1*, *pat1* and *nam7* mutants, the *JEN1* mRNA levels in formic acid are still 50 fold lower than the one measured in lactic acid. Hence, we used a more sensitive technique than GFP fluorescence to try to detect Jen1. We performed western blots, using anti-GFP antibodies, in wild type and *dhh1* mutant grown in glucose, lactic acid or formic acid (Fig. 5B). As expected, the Jen1-GFP protein was detected in no strain in glucose and in all strains in lactic acid. No signal was detected in formic acid, even in the *dhh1* strain, which supported the fact that the jen1 protein was not produced in these conditions (Fig. 5B). Still, these experiments did not exclude the possibility that Jen1 is actually produced but below the detection sensitivity of the method. Indeed, with a simple 50 fold dilution of the lactic acid sample (which roughly mimics the amount of protein that may be expected in the formic acid grown mutant cells, based on the mRNA levels measured in Fig. 1B), the protein became barely detectable (Fig. 5B).

### *JEN1* mRNA is associated with polysomes in the *dhh1* mutant grown in formic acid

**Figure 6:**
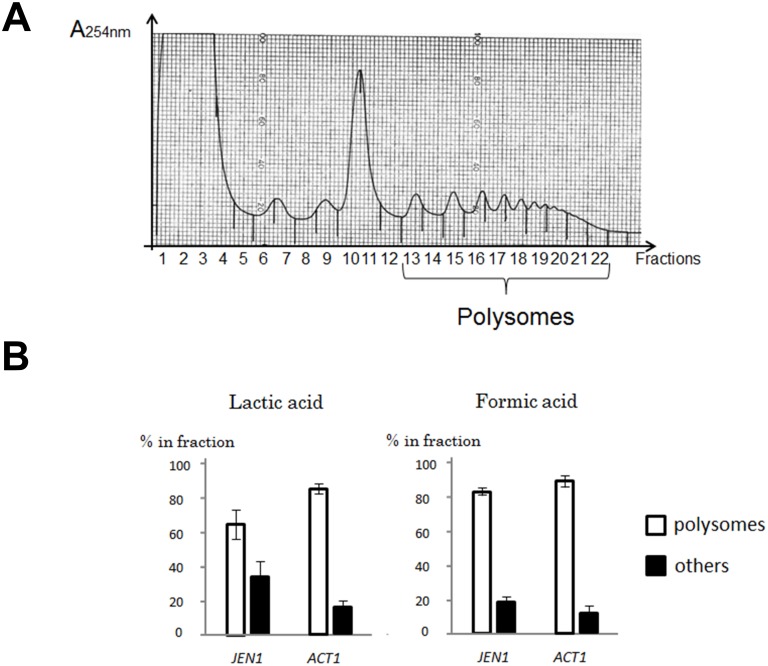
Polysome gradient analyses of *dhh1* mutant cells grown in lactic or formic acids. A: Absorbance profiles along a polysomes gradient obtained with cells grown in lactic acid. The fractions corresponding to the polysomes are indicated. B: the polysomes fractions obtained in acetic and lactic acid were pooled and the percentage of *JEN1* mRNA contained in this fractions compared to the rest of the gradient was quantified by RT-QPCR. In contrast to previous Q-PCR experiments, SCR1 could not be used as a reference because it is not translated. Hence, *ACT1* was used as a control for an mRNA which was actively transcribed in both lactic and formic acids. The experiments were performed three times on biologically independent samples. The difference of *JEN1* abundance in polysomal fractions between lactic and formic acid was not significant, according to a Student test.

To clarify the translational status of the *JEN1* mRNA, we performed polysome gradients in formic acid grown *dhh1* mutant cells (Fig. 6A). Similar experiments were conducted in lactic acid grown *dhh1* cells, as a control for a condition in which *JEN1* mRNA are actively translated. *ACT1* was used as a control for an mRNA which is actively translated in both formic and lactic acid grown cells. The mRNA levels were estimated using real-time quantitative PCR. Because the *JEN1* mRNA levels were very low in formic acid, we had to pool all the fractions corresponding to polysomes on one hand and all the other fractions on the other hand, and make a rough estimate of the percentage of *ACT1* or *JEN1* mRNAs present in each of the two categories of fractions (Fig. 6B). As expected, *JEN1* mRNAs were enriched in the polysome fractions in cells grown in lactic acid. Interestingly, it was also clearly enriched in the polysomes fractions in cells grown in formic acid. These experiments indicated that, although we could not detect any Jen1 activity, the *JEN1* mRNAs, which accumulate in formic acid in the absence of Dhh1, are associated with polysomes.

### Genome-wide analyses of the *dhh1* role in metabolic adaptation

**Figure 7:**
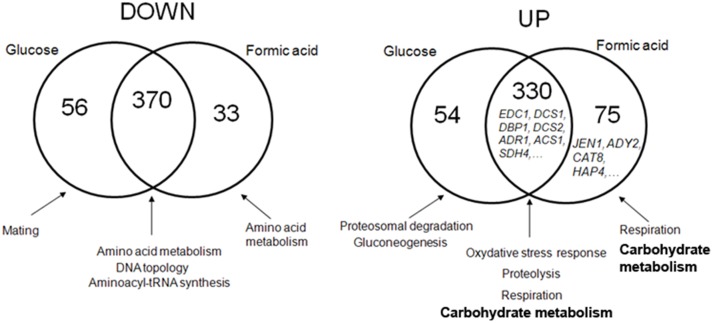
Transcriptome analyses of Dhh1 impact on gene expression. Venn diagram representing the overlap of down (left) and up (right) regulation effects in a dhh1 mutant, grown either in glucose or in formic acid. The main functional categories enriched in each group are indicated. They were determined using the FUNSPEC web tool (funspec.med.utoronto.ca/). The complete set of genes in each category, together with their functional annotation, can be found in Table S1.

To highlight the role of Dhh1 in the gene regulation associated with carboxylic acids and non fermentative growth conditions, we performed DNA microarray analyses of the transcriptome of yeast wild-type and *dhh1* mutant cells, grown in glucose or shifted from glucose to formic acid 0.5%, pH 5.0, for 4 hours. About 920 genes were identified as being significantly up or down regulated in the *dhh1* mutant compared with the wild-type, in at least one of the two tested conditions (Fig. 7). The mRNAs which amounts increased in the mutant were mostly involved in proteasomal and vacuolar proteolysis, respiration, oxidative and general stress responses and carbohydrate metabolism. The mRNA which steady-state decreased in the mutant were involved in ammonia and amino acid metabolism (including most of the corresponding transcriptional regulators), DNA topology and the maintenance and silencing of telomeres, aminoacyl-tRNA synthesis, translational elongation and mating (Fig. 7). About 75% of these effects were independent of the carbon source, i.e. they were found both in glucose and formic acid. Among the genes that accumulated independently of the carbon source, we found the previously identified targets of Dhh1: *EDC1*, *COX17* [25], [44] and *SDH4* [47]. Also, the decrease in expression of genes involved in mating and in tRNA metabolism is reminiscent of the roles of Dhh1 in Ste12 induction [46] and tRNA maturation [51], respectively. Interestingly, several genes involved in mRNA decay were up- (*EDC1*, *EDC2*, *DCS1*, *DCS2*, *PUF3*, *PUF2*) or down- (*POP1* and the subunits of the CCR4-NOT complex *CAF16*, *CAF4* and *NOT3*) regulated in the mutant, suggesting the existence of feedback controls between the activity of Dhh1 and the components of the mRNA degradation pathways. Moreover, some translation regulators exhibited increased (*SRO9*, *PET122*, *PPQ1*, *SUI1*, *CBP6*) or decreased (*TPA1*, *RPS31*, *GCN1*, *RBG2*, *MDM38*, *GCN3*, *ECM32*) expression in the *dhh1* mutant. Intriguingly enough, many genes encoding RNA helicases (*SLH1*, *BRR2*, *DED1*, *DBP1*) and telomeric DNA helicases (Table S1) showed significant expression changes in the mutant.

**Figure 8:**
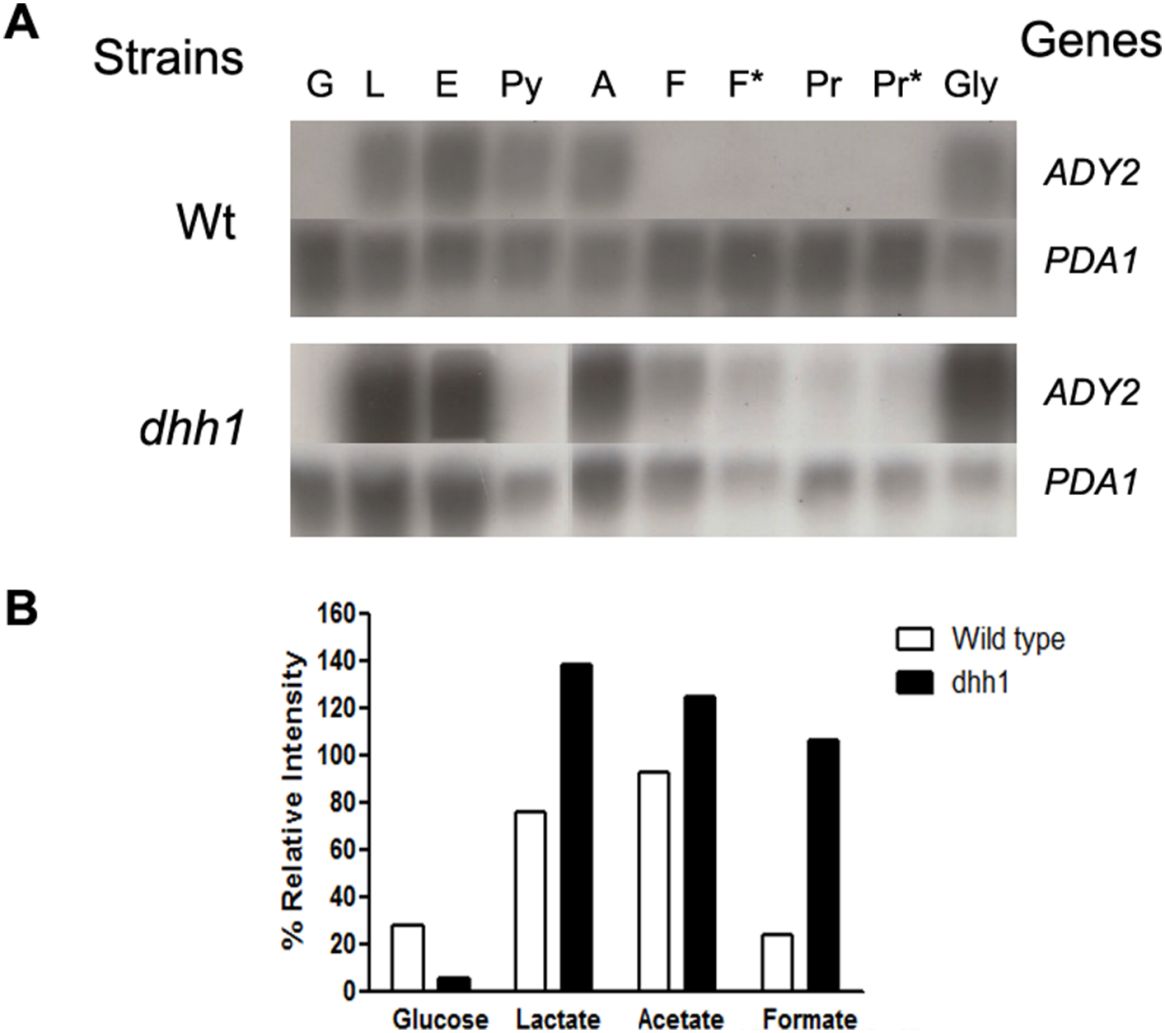
*ADY2* expression profile. A- Transcription analyses of *ADY2* in *S. cerevisiae* W303-1A and *dhh1* cells. Total RNA was isolated from YNB Glucose 2%-grown cells, collected at mid exponential phase, and after induction for 4 or 6 hours in different non-fermentable carbon sources: G – glucose; L – lactic acid (4 hours); E – ethanol (4 hours); Py – pyruvic acid (4 hours); A – Acetic acid (4 hours); F – formic acid (4 hours); F* – formic acid (6 hours); Pr – propionic acid (4 hours); Pr* – propionic acid (6 hours); Gly – glycerol (4 hours). An internal *ADY2* fragment was used as probe. *PDA1* was used as a reference, for relatively constant transcription. B- Densiometry analysis of *ADY2* Northern blots was performed on scanned films using ImageJ gel analysis tool (public domain NIH Image program (developed at the U.S. National Institutes of Health and available on the Internet at http://rsb.info.nih.gov/nih-image/). Absolute intensities were calculated for both *ADY2* and the *PDA1* control. Relative intensities were calculated for each experimental band by normalizing the absolute intensity to the corresponding control intensity.

**Figure 9:**
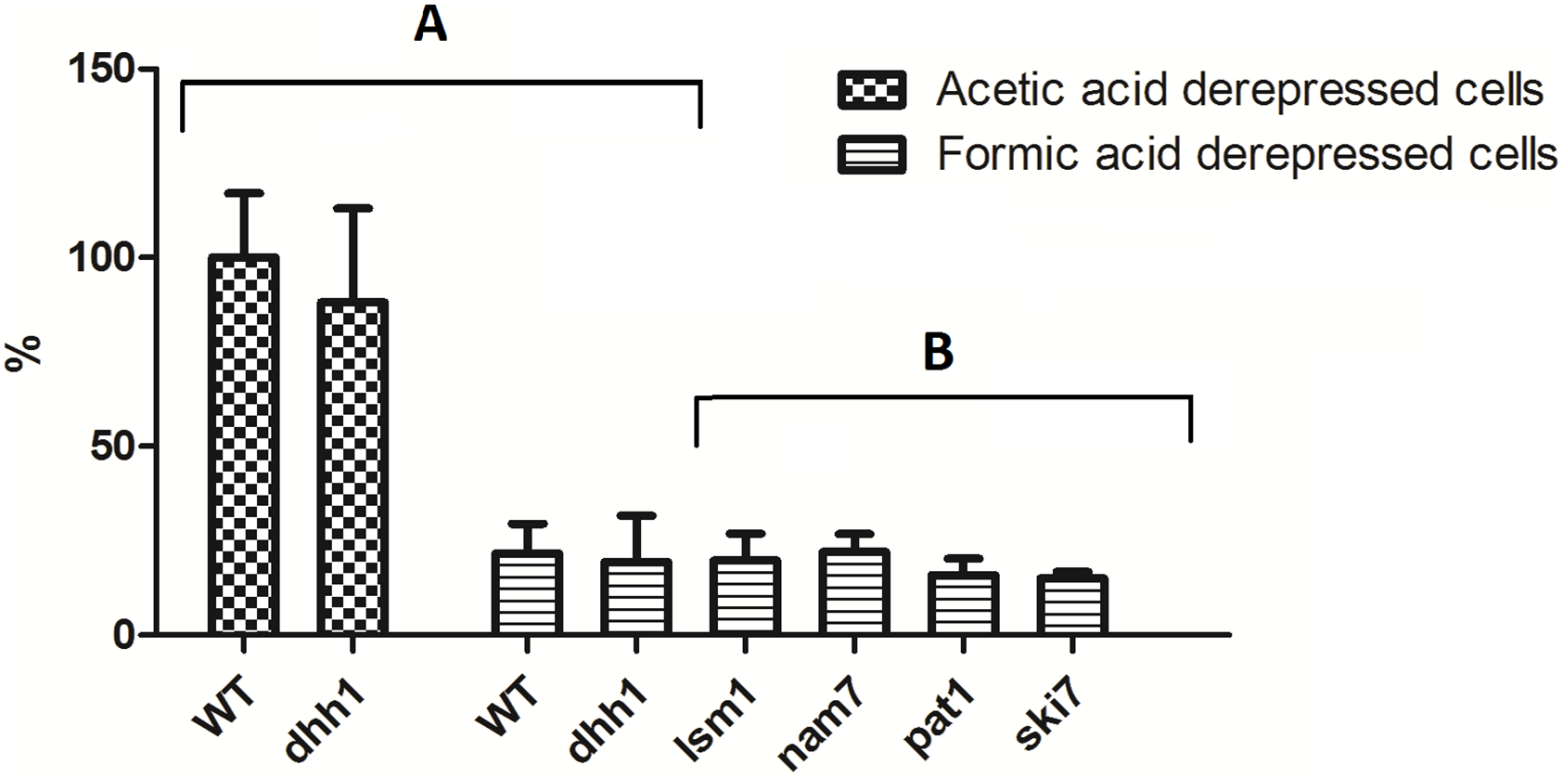
Transport activity of Ady2 in *S. cerevisiae* W303-1A strains: wild-type, *dhh1*, *lsm1*, *ski7*, *pat1* and *nam7*. The results are percentages of initial activities of 2 mM [^14^C] acetic acid uptake, pH 5.0. Cells were grown in YNB glucose and derepressed in YNB acetic acid or YNB formic acid. Wild-type and *dhh1* YNB acetic acid derepressed cells were used as a control.

Finally, microarray results confirmed the accumulation of the *JEN1* mRNA in the mutant strain, only in the presence of formic acid, as previously that was described in this work. Then, we focused our attention on the genes which, like *JEN1*, are repressed in the presence of glucose and induced by acetate [7] (Table S1). We found that 32 of these genes behaved like *JEN1* in the *dhh1* mutant. This is for instance the case of the other carboxylic acid transporter, *ADY2*, of *CAT8*, the transcriptional regulator of *JEN1*, and of the positive regulator of respiratory gene expression, *HAP4*. Northern blot analyses confirmed that the *ADY2* mRNA indeed accumulated in the *dhh1* mutant only in the presence of formic acid (Fig. 8). Additionally, transport activity experiments in formic acid derepressed cells showed that the accumulation of *ADY2* mRNA did not produce detectable amounts of Ady2 protein, nor in *dhh1* mutant nor in the other mutants tested in this work, similar to what was found for Jen1 (Fig. 9). These results suggest that the post-transcriptional regulation that we characterized for *JEN1* is shared by several other genes involved in carbon source metabolism. Surprisingly, several genes that were known to be similarly subjected to glucose repression already accumulated in the *dhh1* mutant in the presence of glucose (Table S1). This is for instance the case of *ADR1*, a transcription factor which collaborates with *CAT8* under non fermentative growth conditions. Noteworthy, *ADR1* mRNA had been already shown to be post-transcriptionally down-regulated by the non-sense mediated mRNA decay machinery and the decapping enzyme Dcp1 in presence of glucose [66]. This raises the interesting hypothesis that the balance between transcriptional and post-transcriptional regulations ensuring glucose catabolic repression may largely differ from one gene to another.

## Discussion

Jen1 is localized at the plasma membrane of *S. cerevisiae* cells and it is involved in the transport of lactic, pyruvic, acetic and propionic acids. This permease is induced in the presence of non-fermentable carbon and energy sources, like lactic and pyruvic acids and its expression is undetectable in the presence of glucose, formic or propionic acids [4], [67]. The disruption of the RNA helicase encoding gene *DHH1* attenuated growth on acetic acid. Dhh1 was known to participate in the mRNA cycle [25] controlling, together with the Pat1-Lsm complex, the balance between translation and mRNA degradation by inhibiting translation initiation, targeting mRNAs to the P-bodies and contributing to the recruitment of the decapping machinery [68]. In this work, we showed that Dhh1 in particular, and the decapping complex in general, have roles in the post-transcriptional regulation of *JEN1* expression, which depend on carbon source. In the absence of Dhh1, Pat1 or Lsm1, *JEN1* mRNAs accumulated in formic acid and associated with polysomes, although we could not detect the translated functional protein. Hence, the translational status of *JEN1* mRNAs in these conditions remains an open question. The same phenomena occurred in a mutant for Nam7/Upf1, which is an important actor of the NMD pathway. Additionally, we confirmed that the half-lives of the *JEN1* mRNA actually increased in the absence of Dhh1, but not in the *nam7* mutant. In contrast, in acetic acid, the inactivation of Pat1 or Dhh1 had a negative effect on *JEN1* mRNA expression. Our microarray experiments suggest that other key genes of metabolic adaptation, like the transcription factor encoding gene *CAT8* or the acetate transporter encoding gene *ADY2* (Fig. 8), may encounter similar regulations.

**Figure 10:**
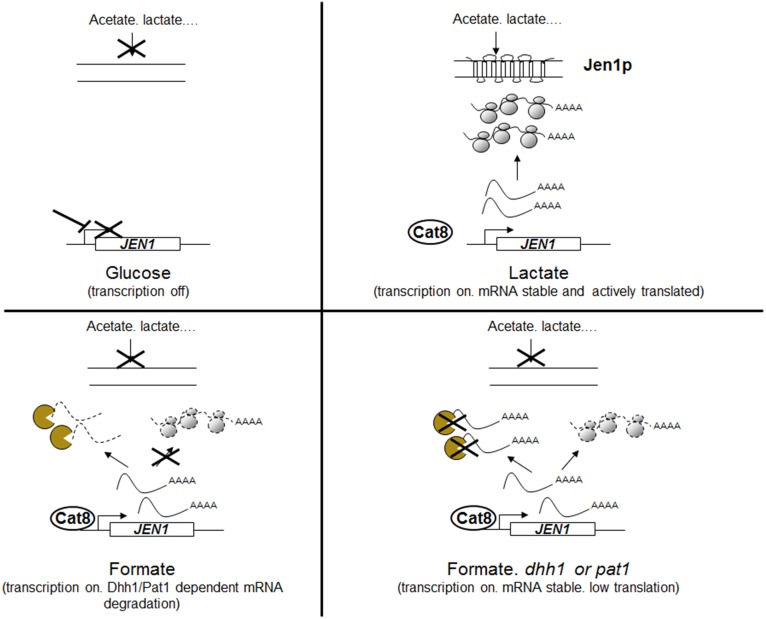
Model for *JEN1* expression regulation. This model is inferred from the data presented in this work. Briefly, in glucose, *JEN1* is transcriptionally silent. In lactate or acetate, *JEN1* is transcriptionally activated by Cat8 and *JEN1* mRNA are actively translated, which results in the accumulation of Jen1 in the plasma membrane and in an active transport of carboxylic acids. In formic acid, *JEN1* is transcriptionally active, but the *JEN1* mRNAs are targeted to degradation in P-bodies and therefore barely detectable by northern blots. In the absence of Dhh1 or Pat1, *JEN1* mRNA are no more degraded but still the Jen1 protein was not detectable, which result in an accumulation of mRNA with no or very few Jen1 protein in the membrane and no or very few active transport of carboxylic acids. Our data cannot tell if this low protein level is due to the low RNA level or to actual translation inhibition.

Hence, the model that we can draw from our results and from previous studies is the following (Fig. 10). In glucose, *JEN1* is transcriptionally silent, as described previously. In lactic, its transcription is induced by Cat8 and Adr1, which results in high levels of Jen1 protein. In formic acid, the glucose transcriptional repression is also released, but *JEN1* mRNAs are rapidly degraded. This degradation requires Dhh1, Pat1 and Lsm1, which are known to collaborate in the activation of decapping and 5′-3′ mRNA decay, but not Ski7, which is involved in the 3′-5′ degradation of cytoplasmic mRNA by the exosome. Notably, the stability of the *JEN1* mRNA increase in the *dhh1* mutant was only two fold, when its accumulation was about 6 fold, suggesting additional levels of controls of Dhh1 on *JEN1* mRNA steady-state. This accumulation of *JEN1* mRNA in formic acid is also dependent on the presence of Nam7, but Nam7 does not act at the level of *JEN1* mRNA stability. *NAM7/UPF1* is involved in the NMD pathway which degrades aberrant mRNAs exhibiting a premature stop codon and “normal” mRNAs which present particular features (long 3′UTRs, alternative translation initiation sites, upstream ORFs) [69], reviewed in [31]. However, our results suggest that *JEN1* mRNA is not a target of Nam7. One possibility is that Nam7 acts indirectly on *JEN1* expression by regulating the levels of a transcriptional regulator of *JEN1* in formic acid.

In acetic acid, the regulation of *JEN1* seems to be totally different. In the wild type, the *JEN1* mRNA is highly expressed. Mutations of *DHH1* or *PAT1* decreased this expression level (Fig. 1 and 2). GFP-fusion experiments showed that the *JEN1* mRNAs are translated in the *dhh1* mutant but that this lower level of mRNA expression resulted in a lower permease activity, as measured by lactate transport assays (Fig. S2 in File S1). These observations may explain the slow-growth phenotype of the *dhh1* mutant in acetic acid. This effect on the mRNA levels of *JEN1* in acetic acid was independent from Nam7 (Fig. 2). The fact that the inactivation of a degradation pathway can lead to a decrease in gene expression may seem counter intuitive. It was shown recently that the inactivation of the cytoplasmic 5′-3′ exonuclease Xrn1 or of the decapping enzyme Dcp2 leads to accumulation of long non coding RNAs (lncRNAs), some of which being located in the promoter or in antisense position of coding genes [70], [71]. In some cases, this accumulation can lead to the transcriptional silencing of the overlapping genes. This system seems to preferentially target inducible genes, as for instance the *GAL* system [70], [72]. The *JEN1* genomic region has been shown to be able to produce two stable unannotated transcripts (SUTs) [73] in sense and antisense positions (www.yeastgenome.org). Moreover, it overlaps with one large Xrn1 sensitive lncRNA (XUT) antisense to the *JEN1* mRNA sequence [71] (www.yeastgenome.org). Therefore, it was tempting to speculate that the negative effects of Dhh1 and Pat1 deletion on *JEN1* expression in acetic acid could be mediated by an accumulation of one or several of these intergenic or antisense lncRNAs. Northern blot analyses of the three non coding RNAs overlapping the *JEN1* locus could not show any difference of expression between the wild type and the *dhh1* mutant grown in acetic acid (data not shown). This suggested that Dhh1 does not act on *JEN1* expression in acetic acid by degrading overlapping transcripts. This is consistent with previous observations that Dhh1 and Pat1 had no role in the transcriptional silencing by the accumulation of lncRNAs [70].

More generally, we pointed out about 900 potential targets for Dhh1, which are involved in many, different cellular pathways. These results emphasized the large role of Dhh1 in gene expression regulation. Still, this number (about 15% of the genes) is relatively small, considering that Dhh1 participates to a general mRNA degradation pathway. Interestingly, in trypanosomes, microarray analyses of *dhh1* mutants suggested that it controls the expression of only 1% of the genes, several of them being involved specifically in developmental processes [74], [75]. More recently, CLIP-seq experiments have shown that Dhh1 was able to bind about 300 mRNAs in standard growth conditions [76]. Our microarray results and the model of *JEN1* regulation discussed above support the idea that, besides its general role in the global cytoplasmic mRNA decay, Dhh1, like Xrn1 or Dcp2 [70]–[72] may have more specific roles in the post-transcriptional and/or transcriptional regulation of some genes, in response to environmental stimuli. Moreover, our list of genes whose expression is affected in the *dhh1* deletion strain provides explanations for the various phenotypes reported for *DHH1* mutations, including defects in G1/S checkpoint recovery, filamentous growth, stress responses, membrane asymmetry, sporulation, ion homeostasis, apoptosis, vacuolar trafficking, ethanol, 2-deoxyglucose and zinc resistance [41], [46], [77]–[86]. However, the interpretation of these mRNA steady-state measurements in terms of direct and indirect effects is not straightforward, since Dhh1 impacts on the expression of a large number of transcriptional and post-transcriptional regulators and of their target genes (Table S1). For instance, the expression of the transcription factor encoding gene *WAR1* (involved in weak acid resistance) and of its main target gene *PDR12* decreased in the *dhh1* mutant. Noteworthy, the level of expression of *DHH1* increased in a *WAR1* gain of function mutant [87]. Similarly, Dhh1 apparently controls the level of expression of several proteins regulating mRNA stability, including for instance *PUF2* and *PUF3*. Some PUF proteins have been shown to promote mRNA decay depending on Dhh1 [88], [89]. This suggests a complex interplay between transcriptional and post-transcriptional effects, with regulatory feedbacks between them. Clearly, further genome-wide mRNA stability and proteome studies of the *dhh1* mutant will be required to decipher the global regulatory roles of Dhh1.

In conclusion, this study revealed that he regulation of *JEN1*, *ADY2* and possibly many other mRNA in carboxylic acids is much more complex than a simple relieve of glucose repression, and that the mechanisms which control this expression considerably vary from one carbon source to another.

## Supporting Information

File S1 **Figures S1 and S2.** Figure S1. Representative growth curves of wild-type and *dhh1* cells grown in YNB glucose 2% (A) and in YP acetic acid 0.5% (B) media. Figure S2. Transport activity and subcellular localization of *Jen1::GFP* in *S. cerevisiae* W303-1A strains. A – Percentages of initial activities of 2 mM lactic acid uptake, at pH 5.0, in cells grown in YNB glucose and derepressed in YNB acetic acid 0.5%, pH 6.0. B – Wild-type and *dhh1* cells harboring Jen1-GFP were used to monitor Jen1 expression after growth in YNB glucose and derepression in YNB acetic acid 0.5% pH 6.0 for 6 hours or YNB lactic acid 0.5% pH 5.0 for 4 hours.
(DOCX)

Table S1 **Lists of genes with significant expression variations.** The criteria used to select these genes can be found in the material and methods. There are three sheets corresponding to the following categories: gene changing expression 1- only in formic acid, 2- only in glucose or 3- in both glucose and formic acid. Column 1: ORF ID, column 2: fold change in acetate compared with glucose (data from Paiva *et al*., Yeast 2004), Column 3: average log of fold change (mutant/wild-type) in glucose, Column 4: average log of fold change (mutant/wild-type) in formic acid, Column 5: gene name, Column 6: functional annotation taken from the SGD.
(XLS)

Table S2 **Complete microarray results.** Column 1: ORF ID. Column 2 to 4: Log2 of normalized fluorescence ratios (*dhh1* mutant/wild-type) for the 4 experiments (two growth conditions in duplicate).
(XLS)

## References

[1] Casal M, Paiva S, Queiros O, Soares-Silva I (2008) Transport of carboxylic acids in yeasts. FEMS Microbiol Rev 32: 974–994. 18759742 10.1111/j.1574-6976.2008.00128.x

[2] Cassio F, Leao C, van Uden N (1987) Transport of lactate and other short-chain monocarboxylates in the yeast Saccharomyces cerevisiae. Appl Environ Microbiol 53: 509–513. 3034152 10.1128/aem.53.3.509-513.1987 PMC203697

[3] Casal M, Blazquez MA, Gamo FJ, Gancedo C, Leao C (1995) Lack of lactate-proton symport activity in pck1 mutants of Saccharomyces cerevisiae. FEMS Microbiol Lett 128: 279–282. 7781975 10.1111/j.1574-6968.1995.tb07536.x

[4] Casal M, Paiva S, Andrade RP, Gancedo C, Leao C (1999) The lactate-proton symport of *Saccharomyces cerevisiae* is encoded by *JEN1* . J Bacteriol 181: 2620–2623. 10198029 10.1128/jb.181.8.2620-2623.1999 PMC93691

[5] Casal M, Cardoso H, Leao C (1996) Mechanisms regulating the transport of acetic acid in Saccharomyces cerevisiae. Microbiology 142 (Pt 6): 1385–1390. 10.1099/13500872-142-6-1385 8704978

[6] Makuc J, Paiva S, Schauen M, Kramer R, Andre B, et al (2001) The putative monocarboxylate permeases of the yeast Saccharomyces cerevisiae do not transport monocarboxylic acids across the plasma membrane. Yeast 18: 1131–1143. 11536335 10.1002/yea.763

[7] Paiva S, Devaux F, Barbosa S, Jacq C, Casal M (2004) Ady2p is essential for the acetate permease activity in the yeast *Saccharomyces cerevisiae* . Yeast 21: 201–210. 14968426 10.1002/yea.1056

[8] Paiva S, Althoff S, Casal M, Leao C (1999) Transport of acetate in mutants of *Saccharomyces cerevisiae* defective in monocarboxylate permeases. FEMS Microbiol Lett 170: 301–306. 9933925 10.1111/j.1574-6968.1999.tb13387.x

[9] Strahl-Bolsinger S, Tanner W (1993) A yeast gene encoding a putative RNA helicase of the “DEAD”-box family. Yeast 9: 429–432. 8511971 10.1002/yea.320090414

[10] Presnyak V, Coller J (2013) The DHH1/RCKp54 family of helicases: an ancient family of proteins that promote translational silencing. Biochim Biophys Acta 1829: 817–823. 23528737 10.1016/j.bbagrm.2013.03.006 PMC3661697

[11] Maekawa H, Nakagawa T, Uno Y, Kitamura K, Shimoda C (1994) The ste13+ gene encoding a putative RNA helicase is essential for nitrogen starvation-induced G1 arrest and initiation of sexual development in the fission yeast Schizosaccharomyces pombe. Mol Gen Genet 244: 456–464. 8078473 10.1007/BF00583896

[12] Ladomery M, Wade E, Sommerville J (1997) Xp54, the Xenopus homologue of human RNA helicase p54, is an integral component of stored mRNP particles in oocytes. Nucleic Acids Res 25: 965–973. 9023105 10.1093/nar/25.5.965 PMC146530

[13] de Valoir T, Tucker MA, Belikoff EJ, Camp LA, Bolduc C, et al (1991) A second maternally expressed Drosophila gene encodes a putative RNA helicase of the “DEAD box” family. Proc Natl Acad Sci U S A 88: 2113–2117. 1900936 10.1073/pnas.88.6.2113 PMC51179

[14] Akao Y, Marukawa O, Morikawa H, Nakao K, Kamei M, et al (1995) The rck/p54 candidate proto-oncogene product is a 54-kilodalton D-E-A-D box protein differentially expressed in human and mouse tissues. Cancer Res 55: 3444–3449. 7614484

[15] Seto M, Yamamoto K, Takahashi T, Ueda R (1995) Cloning and expression of a murine cDNA homologous to the human RCK/P54, a lymphoma-linked chromosomal translocation junction gene on 11q23. Gene 166: 293–296. 8543178 10.1016/0378-1119(95)00559-5

[16] Navarro RE, Shim EY, Kohara Y, Singson A, Blackwell TK (2001) cgh-1, a conserved predicted RNA helicase required for gametogenesis and protection from physiological germline apoptosis in *C. elegans* . Development 128: 3221–3232. 11546739 10.1242/dev.128.17.3221

[17] Sheth U, Parker R (2003) Decapping and decay of messenger RNA occur in cytoplasmic processing bodies. Science 300: 805–808. 12730603 10.1126/science.1082320 PMC1876714

[18] Wickens M, Goldstrohm A (2003) Molecular biology. A place to die, a place to sleep. Science 300: 753–755. 12730589 10.1126/science.1084512

[19] Anderson P, Kedersha N (2006) RNA granules. J Cell Biol 172: 803–808. 16520386 10.1083/jcb.200512082 PMC2063724

[20] Eulalio A, Behm-Ansmant I, Izaurralde E (2007) P bodies: at the crossroads of post-transcriptional pathways. Nat Rev Mol Cell Biol 8: 9–22. 17183357 10.1038/nrm2080

[21] Parker R, Sheth U (2007) P bodies and the control of mRNA translation and degradation. Mol Cell 25: 635–646. 17349952 10.1016/j.molcel.2007.02.011

[22] Olszewska M, Bujarski JJ, Kurpisz M (2012) P-bodies and their functions during mRNA cell cycle: Mini-review. Cell Biochemistry and Function 30: 177–182. 22249943 10.1002/cbf.2804

[23] Brengues M, Teixeira D, Parker R (2005) Movement of eukaryotic mRNAs between polysomes and cytoplasmic processing bodies. Science 310: 486–489. 16141371 10.1126/science.1115791 PMC1863069

[24] Coller J, Parker R (2005) General translational repression by activators of mRNA decapping. Cell 122: 875–886. 16179257 10.1016/j.cell.2005.07.012 PMC1853273

[25] Balagopal V, Parker R (2009) Polysomes, P bodies and stress granules: states and fates of eukaryotic mRNAs. Curr Opin Cell Biol 21: 403–408. 19394210 10.1016/j.ceb.2009.03.005 PMC2740377

[26] Liu J, Rivas FV, Wohlschlegel J, Yates JR 3rd, Parker R, et al (2005) A role for the P-body component GW182 in microRNA function. Nat Cell Biol 7: 1261–1266. 16284623 10.1038/ncb1333 PMC1804202

[27] Pillai RS, Bhattacharyya SN, Artus CG, Zoller T, Cougot N, et al (2005) Inhibition of translational initiation by Let-7 MicroRNA in human cells. Science 309: 1573–1576. 16081698 10.1126/science.1115079

[28] Unterholzner L, Izaurralde E (2004) SMG7 acts as a molecular link between mRNA surveillance and mRNA decay. Mol Cell 16: 587–596. 15546618 10.1016/j.molcel.2004.10.013

[29] Sheth U, Parker R (2006) Targeting of aberrant mRNAs to cytoplasmic processing bodies. Cell 125: 1095–1109. 16777600 10.1016/j.cell.2006.04.037 PMC1858659

[30] Beliakova-Bethell N, Beckham C, Giddings TH Jr, Winey M, Parker R, et al (2006) Virus-like particles of the Ty3 retrotransposon assemble in association with P-body components. RNA 12: 94–101. 16373495 10.1261/rna.2264806 PMC1370889

[31] Parker R (2012) RNA Degradation in Saccharomyces cerevisae. Genetics 191: 671–702. 22785621 10.1534/genetics.111.137265 PMC3389967

[32] Balagopal V, Fluch L, Nissan T (2012) Ways and means of eukaryotic mRNA decay. Biochimica et Biophysica Acta (BBA) - Gene Regulatory Mechanisms 1819: 593–603. 22266130 10.1016/j.bbagrm.2012.01.001

[33] Carroll JS, Munchel SE, Weis K (2011) The DExD/H box ATPase Dhh1 functions in translational repression, mRNA decay, and processing body dynamics. The Journal of Cell Biology 194: 527–537. 21844211 10.1083/jcb.201007151 PMC3160580

[34] Hata H, Mitsui H, Liu H, Bai Y, Denis CL, et al (1998) Dhh1p, a putative RNA helicase, associates with the general transcription factors Pop2p and Ccr4p from *Saccharomyces cerevisiae* . Genetics 148: 571–579. 9504907 10.1093/genetics/148.2.571 PMC1459828

[35] Coller JM, Tucker M, Sheth U, Valencia-Sanchez MA, Parker R (2001) The DEAD box helicase, Dhh1p, functions in mRNA decapping and interacts with both the decapping and deadenylase complexes. RNA 7: 1717–1727. 11780629 10.1017/s135583820101994x PMC1370212

[36] Franks TM, Lykke-Andersen J (2008) The control of mRNA decapping and P-body formation. Mol Cell 32: 605–615. 19061636 10.1016/j.molcel.2008.11.001 PMC2630519

[37] Maillet L, Collart MA (2002) Interaction between Not1p, a component of the Ccr4-not complex, a global regulator of transcription, and Dhh1p, a putative RNA helicase. J Biol Chem 277: 2835–2842. 11696541 10.1074/jbc.M107979200

[38] Kshirsagar M, Parker R (2004) Identification of Edc3p as an enhancer of mRNA decapping in Saccharomyces cerevisiae. Genetics 166: 729–739. 15020463 10.1093/genetics/166.2.729 PMC1470743

[39] Sharif H, Ozgur S, Sharma K, Basquin C, Urlaub H, et al.. (2013) Structural analysis of the yeast Dhh1-Pat1 complex reveals how Dhh1 engages Pat1, Edc3 and RNA in mutually exclusive interactions. Nucleic Acids Res. 10.1093/nar/gkt600 PMC3783180 23851565

[40] Fischer N, Weis K (2002) The DEAD box protein Dhh1 stimulates the decapping enzyme Dcp1. EMBO J 21: 2788–2797. 12032091 10.1093/emboj/21.11.2788 PMC126031

[41] Tseng-Rogenski SS, Chong JL, Thomas CB, Enomoto S, Berman J, et al (2003) Functional conservation of Dhh1p, a cytoplasmic DExD/H-box protein present in large complexes. Nucleic Acids Res 31: 4995–5002. 12930949 10.1093/nar/gkg712 PMC212811

[42] Wyers F, Minet M, Dufour ME, Vo LT, Lacroute F (2000) Deletion of the PAT1 gene affects translation initiation and suppresses a PAB1 gene deletion in yeast. Mol Cell Biol 20: 3538–3549. 10779343 10.1128/mcb.20.10.3538-3549.2000 PMC85646

[43] Sweet T, Kovalak C, Coller J (2012) The DEAD-box protein Dhh1 promotes decapping by slowing ribosome movement. PLoS Biol 10: e1001342. 22719226 10.1371/journal.pbio.1001342 PMC3373615

[44] Muhlrad D, Parker R (2005) The yeast EDC1 mRNA undergoes deadenylation-independent decapping stimulated by Not2p, Not4p, and Not5p. EMBO J 24: 1033–1045. 15706350 10.1038/sj.emboj.7600560 PMC554118

[45] Ka M, Park YU, Kim J (2008) The DEAD-box RNA helicase, Dhh1, functions in mating by regulating Ste12 translation in Saccharomyces cerevisiae. Biochem Biophys Res Commun 367: 680–686. 18182159 10.1016/j.bbrc.2007.12.169

[46] Park YU, Hur H, Ka M, Kim J (2006) Identification of translational regulation target genes during filamentous growth in Saccharomyces cerevisiae: regulatory role of Caf20 and Dhh1. Eukaryot Cell 5: 2120–2127. 17041186 10.1128/EC.00121-06 PMC1694813

[47] Pedro-Segura E, Vergara SV, Rodriguez-Navarro S, Parker R, Thiele DJ, et al (2008) The Cth2 ARE-binding protein recruits the Dhh1 helicase to promote the decay of succinate dehydrogenase SDH4 mRNA in response to iron deficiency. J Biol Chem 283: 28527–28535. 18715869 10.1074/jbc.M804910200 PMC2568921

[48] Liu H, Kiledjian M (2005) Scavenger decapping activity facilitates 5′ to 3′ mRNA decay. Mol Cell Biol 25: 9764–9772. 16260594 10.1128/MCB.25.22.9764-9772.2005 PMC1280280

[49] Tseng SS, Weaver PL, Liu Y, Hitomi M, Tartakoff AM, et al (1998) Dbp5p, a cytosolic RNA helicase, is required for poly(A)+ RNA export. EMBO J 17: 2651–2662. 9564047 10.1093/emboj/17.9.2651 PMC1170606

[50] Snay-Hodge CA, Colot HV, Goldstein AL, Cole CN (1998) Dbp5p/Rat8p is a yeast nuclear pore-associated DEAD-box protein essential for RNA export. EMBO J 17: 2663–2676. 9564048 10.1093/emboj/17.9.2663 PMC1170607

[51] Hurto RL, Hopper AK (2011) P-body components, Dhh1 and Pat1, are involved in tRNA nuclear-cytoplasmic dynamics. RNA 17: 912–924. 21398402 10.1261/rna.2558511 PMC3078740

[52] Checkley MA, Nagashima K, Lockett SJ, Nyswaner KM, Garfinkel DJ (2010) P-body components are required for Ty1 retrotransposition during assembly of retrotransposition-competent virus-like particles. Mol Cell Biol 30: 382–398. 19901074 10.1128/MCB.00251-09 PMC2798465

[53] Stribinskis V, Ramos KS (2007) Rpm2p, a protein subunit of mitochondrial RNase P, physically and genetically interacts with cytoplasmic processing bodies. Nucleic Acids Res 35: 1301–1311. 17267405 10.1093/nar/gkm023 PMC1851656

[54] Goldstein AL, McCusker JH (1999) Three new dominant drug resistance cassettes for gene disruption in *Saccharomyces cerevisiae* . Yeast 15: 1541–1553. 10514571 10.1002/(SICI)1097-0061(199910)15:14<1541::AID-YEA476>3.0.CO;2-K

[55] Paiva S, Kruckeberg AL, Casal M (2002) Utilization of green fluorescent protein as a marker for studying the expression and turnover of the monocarboxylate permease Jen1p of *Saccharomyces cerevisiae* . Biochem J 363: 737–744. 11964174 10.1042/0264-6021:3630737 PMC1222526

[56] Kruckeberg AL, Ye L, Berden JA, van Dam K (1999) Functional expression, quantification and cellular localization of the Hxt2 hexose transporter of Saccharomyces cerevisiae tagged with the green fluorescent protein. Biochem J 339 (Pt 2): 299–307. PMC1220158 10191260

[57] Sambrook J, Fritsch E.F., and Maniatis T. (1989) Molecular Cloning: A Laboratory Manual; edn n, editor: Cold Spring Harbor Laboratory Press, NY.

[58] Ausubel F, Brent R, Kingston D, Moore D, Seidman JG, Smith, JA SK (1998) In: Current protocols in molecular biology. In: wiley, editor. New York. 4.9.1–4.9.11.

[59] R. Parker DH, S.W Peltz, A Jacobson (1991) Measurement of mRNA decay rates in Saccharomyces cerevisiae. C Guthrie, GR Fink (Eds), Guide to Yeast Genetics and molecular Biology, Methods in exymology. London: Academic Press. 415–423. 10.1016/0076-6879(91)94032-8 2005800

[60] Fardeau V, Lelandais G, Oldfield A, Salin H, Lemoine S, et al (2007) The central role of PDR1 in the foundation of yeast drug resistance. J Biol Chem 282: 5063–5074. 17158869 10.1074/jbc.M610197200

[61] Lemoine S, Combes F, Servant N, Le Crom S (2006) Goulphar: rapid access and expertise for standard two-color microarray normalization methods. BMC Bioinformatics 7: 467. 17059595 10.1186/1471-2105-7-467 PMC1626094

[62] Saeed AI, Sharov V, White J, Li J, Liang W, et al (2003) TM4: a free, open-source system for microarray data management and analysis. Biotechniques 34: 374–378. 12613259 10.2144/03342mt01

[63] Saeed AI, Bhagabati NK, Braisted JC, Liang W, Sharov V, et al (2006) TM4 microarray software suite. Methods Enzymol 411: 134–193. 16939790 10.1016/S0076-6879(06)11009-5

[64] Tusher VG, Tibshirani R, Chu G (2001) Significance analysis of microarrays applied to the ionizing radiation response. Proc Natl Acad Sci U S A 98: 5116–5121. 11309499 10.1073/pnas.091062498 PMC33173

[65] Robinson MD, Grigull J, Mohammad N, Hughes TR (2002) FunSpec: a web-based cluster interpreter for yeast. BMC Bioinformatics 3: 35. 12431279 10.1186/1471-2105-3-35 PMC139976

[66] Taylor R, Kebaara BW, Nazarenus T, Jones A, Yamanaka R, et al (2005) Gene set coregulated by the Saccharomyces cerevisiae nonsense-mediated mRNA decay pathway. Eukaryot Cell 4: 2066–2077. 16339724 10.1128/EC.4.12.2066-2077.2005 PMC1317485

[67] Andrade RP, Casal M (2001) Expression of the lactate permease gene *JEN1* from the yeast *Saccharomyces cerevisiae* . Fungal Genet Biol 32: 105–111. 11352531 10.1006/fgbi.2001.1254

[68] Nissan T, Rajyaguru P, She M, Song H, Parker R (2010) Decapping activators in Saccharomyces cerevisiae act by multiple mechanisms. Mol Cell 39: 773–783. 20832728 10.1016/j.molcel.2010.08.025 PMC2946179

[69] Guan Q, Zheng W, Tang S, Liu X, Zinkel RA, et al (2006) Impact of nonsense-mediated mRNA decay on the global expression profile of budding yeast. PLoS Genet 2: e203. 17166056 10.1371/journal.pgen.0020203 PMC1657058

[70] Geisler S, Lojek L, Khalil AM, Baker KE, Coller J (2012) Decapping of long noncoding RNAs regulates inducible genes. Mol Cell 45: 279–291. 22226051 10.1016/j.molcel.2011.11.025 PMC3278590

[71] van Dijk EL, Chen CL, d’Aubenton-Carafa Y, Gourvennec S, Kwapisz M, et al (2011) XUTs are a class of Xrn1-sensitive antisense regulatory non-coding RNA in yeast. Nature 475: 114–117. 21697827 10.1038/nature10118

[72] Houseley J, Rubbi L, Grunstein M, Tollervey D, Vogelauer M (2008) A ncRNA modulates histone modification and mRNA induction in the yeast GAL gene cluster. Mol Cell 32: 685–695. 19061643 10.1016/j.molcel.2008.09.027 PMC7610895

[73] Xu Z, Wei W, Gagneur J, Perocchi F, Clauder-Munster S, et al (2009) Bidirectional promoters generate pervasive transcription in yeast. Nature 457: 1033–1037. 19169243 10.1038/nature07728 PMC2766638

[74] Kramer S, Queiroz R, Ellis L, Hoheisel JD, Clayton C, et al (2010) The RNA helicase DHH1 is central to the correct expression of many developmentally regulated mRNAs in trypanosomes. J Cell Sci 123: 699–711. 20124414 10.1242/jcs.058511 PMC2823576

[75] Holetz FB, Alves LR, Probst CM, Dallagiovanna B, Marchini FK, et al (2010) Protein and mRNA content of TcDHH1-containing mRNPs in Trypanosoma cruzi. FEBS J 277: 3415–3426. 20629747 10.1111/j.1742-4658.2010.07747.x

[76] Mitchell SF, Jain S, She M, Parker R (2013) Global analysis of yeast mRNPs. Nat Struct Mol Biol 20: 127–133. 23222640 10.1038/nsmb.2468 PMC3537908

[77] Erez O, Kahana C (2002) Deletions of SKY1 or PTK2 in the Saccharomyces cerevisiae trk1Deltatrk2Delta mutant cells exert dual effect on ion homeostasis. Biochem Biophys Res Commun 295: 1142–1149. 12135613 10.1016/s0006-291x(02)00823-9

[78] Westmoreland TJ, Olson JA, Saito WY, Huper G, Marks JR, et al (2003) Dhh1 regulates the G1/S-checkpoint following DNA damage or BRCA1 expression in yeast. J Surg Res 113: 62–73. 12943812 10.1016/s0022-4804(03)00155-0

[79] Bergkessel M, Reese JC (2004) An essential role for the Saccharomyces cerevisiae DEAD-box helicase DHH1 in G1/S DNA-damage checkpoint recovery. Genetics 167: 21–33. 15166134 10.1534/genetics.167.1.21 PMC1470881

[80] Mazzoni C, Mancini P, Verdone L, Madeo F, Serafini A, et al (2003) A truncated form of KlLsm4p and the absence of factors involved in mRNA decapping trigger apoptosis in yeast. Mol Biol Cell 14: 721–729. 12589065 10.1091/mbc.E02-05-0258 PMC150003

[81] Kushner N, Zhang D, Touzjian N, Essex M, Lieberman J, et al (2003) A fragment of anthrax lethal factor delivers proteins to the cytosol without requiring protective antigen. Proc Natl Acad Sci U S A 100: 6652–6657. 12740437 10.1073/pnas.1131930100 PMC164502

[82] Kihara A, Igarashi Y (2004) Cross talk between sphingolipids and glycerophospholipids in the establishment of plasma membrane asymmetry. Mol Biol Cell 15: 4949–4959. 15342785 10.1091/mbc.E04-06-0458 PMC524749

[83] Fujita K, Matsuyama A, Kobayashi Y, Iwahashi H (2006) The genome-wide screening of yeast deletion mutants to identify the genes required for tolerance to ethanol and other alcohols. FEMS Yeast Res 6: 744–750. 16879425 10.1111/j.1567-1364.2006.00040.x

[84] Pagani MA, Casamayor A, Serrano R, Atrian S, Arino J (2007) Disruption of iron homeostasis in Saccharomyces cerevisiae by high zinc levels: a genome-wide study. Mol Microbiol 65: 521–537. 17630978 10.1111/j.1365-2958.2007.05807.x

[85] Ralser M, Wamelink MM, Struys EA, Joppich C, Krobitsch S, et al (2008) A catabolic block does not sufficiently explain how 2-deoxy-D-glucose inhibits cell growth. Proc Natl Acad Sci U S A 105: 17807–17811. 19004802 10.1073/pnas.0803090105 PMC2584745

[86] Banuelos MG, Moreno DE, Olson DK, Nguyen Q, Ricarte F, et al (2010) Genomic analysis of severe hypersensitivity to hygromycin B reveals linkage to vacuolar defects and new vacuolar gene functions in Saccharomyces cerevisiae. Curr Genet 56: 121–137. 20043226 10.1007/s00294-009-0285-3 PMC2886664

[87] Gregori C, Schuller C, Frohner IE, Ammerer G, Kuchler K (2008) Weak organic acids trigger conformational changes of the yeast transcription factor War1 in vivo to elicit stress adaptation. J Biol Chem 283: 25752–25764. 18621731 10.1074/jbc.M803095200

[88] Blewett NH, Goldstrohm AC (2012) A eukaryotic translation initiation factor 4E-binding protein promotes mRNA decapping and is required for PUF repression. Mol Cell Biol 32: 4181–4194. 22890846 10.1128/MCB.00483-12 PMC3457345

[89] Goldstrohm AC, Hook BA, Seay DJ, Wickens M (2006) PUF proteins bind Pop2p to regulate messenger RNAs. Nat Struct Mol Biol 13: 533–539. 16715093 10.1038/nsmb1100

[90] Thomas BJ, Rothstein R (1989) Elevated recombination rates in transcriptionally active DNA. Cell 56: 619–630. 2645056 10.1016/0092-8674(89)90584-9

