# Supplementary material for: Role of the DHH1 Gene in the Regulation of Monocarboxylic Acids Transporters Expression in Saccharomyces cerevisiae
Source: PLoS One. 2014 Nov 3;9(11):e111589. doi: 10.1371/journal.pone.0111589 (PMC4218774; doi:10.1371/journal.pone.0111589)
Supplement: File S1 — Figures S1 and S2. Figure S1. Representative growth curves of wild-type and dhh1 cells grown in YNB glucose 2% (A) and in YP acetic acid 0.5% (B) media. Figure S2. Transport activity and subcellular localization of Jen1::GFP in S. cerevisiae W303-1A strains. A – Percentages of initial activities of 2 mM lactic acid uptake, at pH 5.0, in cells grown in YNB glucose and derepressed in YNB acetic acid 0.5%, pH 6.0. B – Wild-type and dhh1 cells harboring Jen1-GFP were used to monitor Jen1 expression after growth in YNB glucose and derepression in YNB acetic acid 0.5% pH 6.0 for 6 hours or YNB lactic acid 0.5% pH 5.0 for 4 hours. (DOCX) [file pone.0111589.s001.docx]

**
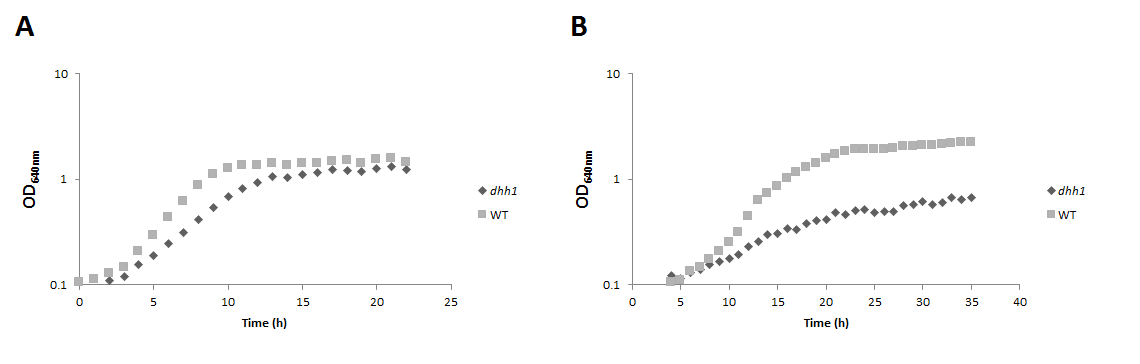
**

**Figure S1**. Representative growth curves of wild-type and *dhh1* cells grown in YNB glucose 2% (A) and in YP acetic acid 0.5% (B) media.


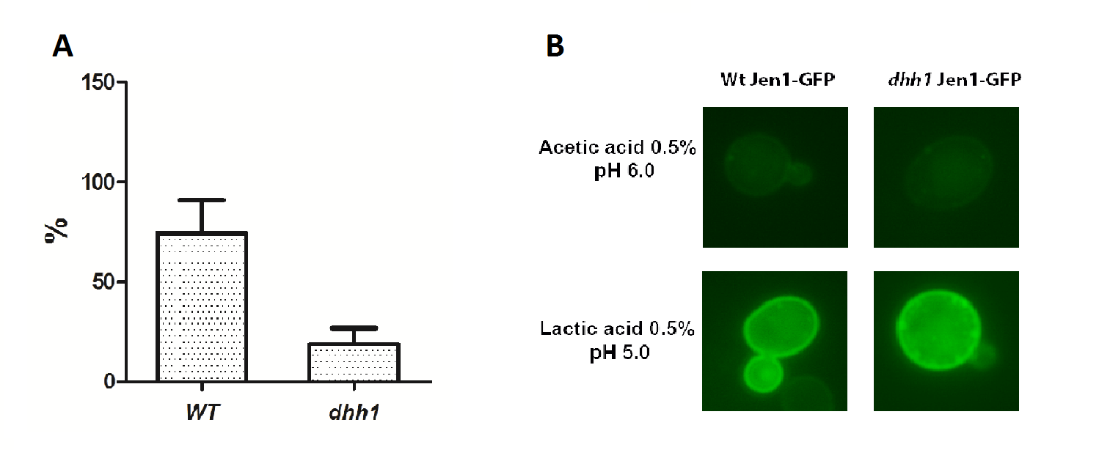


**Figure S2.** Transport activity and subcellular localization of *Jen1::GFP* in *S. cerevisiae* W303-1A strains. A – Percentages of initial activities of 2 mM lactic acid uptake, at pH 5.0, in cells grown in YNB glucose and derepressed in YNB acetic acid 0.5%, pH 6.0. B – Wild-type and *dhh1* cells harboring Jen1-GFP were used to monitor Jen1 expression after growth in YNB glucose and derepression in YNB acetic acid 0.5% pH 6.0 for 6 hours or YNB lactic acid 0.5% pH 5.0 for 4 hours.
